# A unique epigenomic landscape defines the characteristics and differentiation potentials of glioma stem cells

**DOI:** 10.1186/s13059-018-1429-x

**Published:** 2018-04-10

**Authors:** Bing Yao, Peng Jin

**Affiliations:** Department of Human Genetics, Emory University School of Medicine, Atlanta, GA 30322 USA

## Abstract

A new study reveals comprehensive and unique epigenetic properties of glioma stem cells, leading to novel molecular insights and therapeutic potentials toward glioblastoma multiforme treatment.

## Introduction

Glioblastoma multiforme (GBM), also known as glioblastoma, is one of the most aggressive and infiltrative tumors in the central nervous system (CNS). The 5-year survival rate of patients with GBM is less than 5%, and they have a poor prognosis for recovery [1]. Therefore, new, effective therapeutic approaches to treat GBM are urgently needed. Despite substantial progress over the past decades, our understanding of GBM pathology at the molecular level is still largely lacking, owing to its heterogeneous nature with unique genetic and epigenetic alterations [2].

Neurogenesis generates various functional neural cell types from multipotent neural stem cells (NSCs) in the mammalian CNS, which participate in specific neural activities and responses [3]. The concept of the cancer stem cell is based on the fact that a subpopulation of glioma cells share common features with NSCs, including self-renewal and the differentiation potential to glial and neuronal cells [4]. This model explains how cancer stem cells could serve as driving forces of cancer malignancy and recurrence. In vitro*,* cultured glioma stem cells (GSCs) that were derived from primary GBM cells possess many features of NSCs and remarkably have characteristics resembling those of GBM cells, such as genetic aberrations, global transcriptome profiles, and tumorigenic potentials [5]. Therefore, GSCs are an excellent therapeutic target for GBM treatment. The lack of complete and high-quality epigenetic and transcriptome maps in GSCs hinders our understanding of the underlying critical differences at the transcriptional level between malignant GSCs and normal NSCs, as well as the epigenetic machinery involved in regulating their proliferation and differentiation.

In this issue of Genome Biology, Zhou et al. take on this vital challenge to generate comprehensive genome-wide epigenomic profiling for various forms of cytosine modifications of DNA at single-base resolution and signature histone modifications of enhancers and transcriptomes of GSCs isolated from patient-derived xenografts, with NSCs isolated from fetal brains as controls [6].

## Epigenetic dysregulations in GSCs

Covalent modifications of DNA at the 5-carbon position of cytosine, such as 5-methylcytosine (5mC), play significant epigenetic roles in the mammalian brain. 5mC is thought to be a static and irreversible modification. However, the recent discovery that Ten-eleven translocation (TET) proteins, viewed as 5mC “erasers,” catalyze the conversion of 5mC to 5-hydroxymethylcytosine (5hmC), providing a new perspective on the plasticity of cytosine modification. Subsequent studies revealed that TET proteins further oxidize 5hmC to 5-formylcytosine (5fC) and 5-carboxylcytosine (5caC), which are converted to cytosine through DNA repair pathways by thymine-DNA glycosylase (TDG). Cytosine modifications are dynamically and precisely regulated during neurodevelopment and neuronal functions, to spatially and temporally control key gene expression. Thus their dysregulations are linked to many brain disorders, including GBM [7, 8].

In this study, the authors first focused on connecting the expression of DNA modification machinery and the global levels of these modifications in GSCs. There was a notable depletion of certain DNA modifications, such as 5hmC and 5fC, compared with NSCs, indicating a global DNA modification perturbation in GSCs. Interestingly, they found a significant negative correlation of 5mC modification with TET2 and TDG expression, and a positive correlation between TET2 expression and 5fC modification, in GSCs. On the basis of the findings by Zhou et al., it is plausible that TET2 sits at the center stage of maintaining DNA modification homeostasis in normal brain function, and its dysregulation could result in the profound alteration of neuroepigenetic landscaping, contributing to GBM tumorigenesis.

## GSCs display global transcriptome alterations

Transcriptome profiling of GSCs by Zhou et al. identified the upregulation of HOX genes, which potentially account for the GSC proliferation, and the downregulation of genes involved in apoptosis, growth inhibition, and neural development. Also, several highly conserved downregulated miRNAs were observed and potentially linked to patient survival. These findings at the molecular level identify the characteristics of GSCs and provide solid foundations for future mechanistic exploration.

The authors also performed systematic, integrated analyses of genome-wide DNA modification dynamics, enhancer activities, and gene expression changes to define the epigenetic mechanisms that account for the transcriptome alteration in GSCs. While GSCs share a significant portion of enhancers with NSCs, as defined by their histone marks, many GSC-specific active enhancers were identified that potentially link to gene misregulation. These enhancer-shifting patterns indicate that GSCs could lose a set of NSC-specific enhancer footprints and gain a new set of *cis*-regulatory regions to influence tumorigenic transcription ectopically. In addition, global loss of 5mC and 5hmC accompanied by gain of 5fC or 5caC were found in GSCs, which could contribute to gene misregulation by recruiting a distinct set of “readers.” A recent observation suggests that TET2 plays a critical role in further converting 5hmC to 5fC/5caC, as depletion of TET2 results in the accumulation of 5hmC in promoters and gene bodies [7]. The upregulation of TET2 and accumulation of 5fC/5caC seen in this study support this notion. Further research on the molecular mechanisms dictating TET2 differential activities toward 5mC/5hmC or 5hmC/5fC conversion is necessary to confirm this. Nonetheless, these integrated analyses by Zhou et al. highlight the epigenetic causality of GSC-associated transcriptional alteration and demonstrate the significant roles of TET proteins during this process.

## Unique epigenetic profiles associated with GSC differentiation

Asymmetrical differentiation of GSCs could contribute to the tumor mass, and terminal differentiation of these GSCs could eliminate these cell populations for ideal therapeutic outcomes. Epigenetic changes of GSC and NSC differentiation were profiled in parallel, to understand the precise epigenetic alterations during GSC differentiation relative to that of NSCs. While NSC differentiation acquires developmentally programmed DNA modifications during differentiation, the DNA modifications in the GSC genome appear to be more loosely or randomly controlled, which may explain the fact that GSCs possess greater plasticity regarding various endogenous and extracellular differentiation cues for tumorigenesis. Consistent with this, the GSCs with differential TET2 or TET3 expression levels clearly displayed distinctive responses to chemotherapeutic agents, providing important mechanistic insights of current therapeutic challenges. Given the central role of TET proteins in tumorigenesis, it is possible that they may serve as therapeutic targets for GBM patients. Owing to technical challenges and poor antibody quality, the genome-wide distributions of TET proteins in GSCs are still unknown, and it would be important to correlate the binding dynamics of epigenetic machinery with their marks to gain an improved understanding of their molecular mechanisms in order to further therapeutic efforts.

## Conclusions

The present study presents the first detailed characterization of the GSC epigenome, including DNA methylation/demethylation and histone modifications associated with distal regulatory elements. However, the GSCs used in this study were isolated from GBM patient-derived xenografts. The development of single-cell-transcriptome and epigenome analyses will enable the further delineation of epigenomic changes associated with primary GBM tumors and GSCs, as well as tumor organoids. These results will provide further insight into epigenomic alterations associated with GBM and GSCs. More importantly, it will be critical to determine the functionality of causal loci with epigenetic alterations associated with GBM, which may lead to identification of novel therapeutic targets and pathway for GBM.
